# UPDATING ESTIMATES OF DRIVING LIFE EXPECTANCY

**DOI:** 10.1093/geroni/igac059.1986

**Published:** 2022-12-20

**Authors:** Jonathon Vivoda

**Affiliations:** Miami University, Oxford, Ohio, United States

## Abstract

Information is limited about driving life expectancy, and the amount of time between loss of driving ability and death. The most often cited study (by Foley and colleagues, 2002) analyzed data collected in the mid-1990s and used life expectancy estimates to determine survival probabilities. Although that study was well designed, longitudinal data from the Health and Retirement Study (HRS) are now available to use participants’ actual date of death to assess issues related to driving life expectancy. HRS data from 1996-2018 were assessed; only participants who had answered the driving ability question for at least one wave, and had a reported death date were included. The percentages of participants were determined who never reported the ability to drive, always reported the ability to drive, and those who transitioned from driving to non-driving. Time between loss of driving ability and death was calculated among former drivers by subtracting the interview date when respondents reported an inability to drive (after having previously reported being able to drive) from the participants’ date of death. Only about 3% reported never driving, with a nearly even split between those who stopped driving (49%) and those who always drove (48%). Women were more likely to report being a never/former driver (65%) compared to men (36%). Among former drivers, the average time between inability to drive and death was 1312 days (3.59 years), and was significantly longer for women (437 days). Among former drivers, the average age for reporting inability to drive was about 83 years old.

